# Handmade Comal Tortillas in Michoacán: Traditional Practices along the Rural-Urban Gradient

**DOI:** 10.3390/ijerph16173211

**Published:** 2019-09-03

**Authors:** Esperanza Arnés, Marta Astier

**Affiliations:** Centre for Research in Environmental Geography (CIGA), National Autonomous University of Mexico (UNAM), Morelia Campus, Michoacán 58190, Mexico

**Keywords:** maize, food security, agrobiodiversity, traditional food systems, local knowledge, cultural practices, sustainable agriculture

## Abstract

Certain components of global food security continue to be threatened. Globalization has impacted food patterns, leading to greater homogenization of diets and the standardization of processes of food transformation, both in the countryside and in the cities. In Mexico, this has led to a drop in the use of native corn landraces and in the value associated with traditional practices around their growing and the processing and consumption of tortillas. The aim of this work was to analyze the main characteristics of the handmade comal tortilla system along the rural-urban gradient taking into account: (1) The type of seed and production, (2) manufacturing processes, (3) marketing channels and purpose of sales, and (4) perceptions regarding the quality of the product. Research was conducted on 41 handmade tortilla workshops located in rural areas in the Lake Pátzcuaro Basin and in urban and peri-urban areas of a medium-sized city in Michoacán (Mexico). Results showed that the origin of the grain follows a gradient-like pattern: In rural areas, tortillas are made with local and native corn predominate, while in urban contexts most tortillas come from hybrid corn produced in Sinaloa or Jalisco. There is a generalized preference for white tortillas, but blue tortillas are used for personal consumption in rural areas and as a gourmet product in the city. 100% of the rural workshops make their own nixtamal, while almost 50% of the peri-urban and urban businesses buy pre-made nixtamal dough. Surprisingly, 50% of the rural handmade tortilla workshops admit that they add nixtamalized corn flour and/or wheat flour to their tortilla mix. We conclude that not all handmade comal tortillas are produced equally and, although in rural areas traditions are better preserved, these also have contradictions. We also conclude that it is important to promote the revaluation of agrobiodiversity, traditional gastronomy, and food security without sacrificing quality, nutrition, and flavor.

## 1. Introduction

### 1.1. Food Security and Food Patterns

The current global food model is clearly inefficient and harmful, as attested by the world’s 821 million hungry and 672 million obese [1]. These numbers point to a problem that goes beyond the lack of availability or access to food, but rather to its misutilization. Utilization, along with availability, access, and stability is one of the four components that make up the concept of food and nutrition security (food and nutrition security is defined, in a broad sense, as “*when all people at all times have physical, social, and economic access to food, which is safe and consumed in sufficient quantity and quality to meet their dietary needs and food preferences (…)* [2,3].

These maladjustments are the consequence of a change in global food patterns, characterized by an increase in the consumption of meat, dairy, and processed products (high in fats and sugar), and a decrease in cereals, legumes, and vegetables [4,5]. The tendencies of this food transition began to be documented in the 1990s and, were initially linked mainly to urban environments [6]. However, similar patterns can be currently observed in rural areas, and it is increasingly becoming directly related to the socioeconomic level of the population [7,8]. This change in food patterns in the last decades, driven by the demands of globalization, responds to a wide number of complex dynamics that are evident in specific local context [9]. Some authors call it glocalization and it may be related to ignorance about the composition and origin of foods, not valuing traditional cooking techniques, easy access (physical and economically) to processed foods of highly publicized brands and difficult access to healthy foods in low-income areas (the current dichotomy of food-deserts/food-swamps) [10,11].

Mexico is not an exception to this state of affairs, and it lives with the paradox of malnutrition (obesity and hunger) both in rural and urban areas [5,8]. Mexico has the second-highest obesity rate in the world (32.4% of people over 15 years old) and the first rate of child obesity [12]. According to studies, this is due to an increase in the consumption of saturated fats and it coincides in timing with the incorporation of Mexico to the North American Free Trade Agreement (NAFTA) in 1994 [13]. Moreover, globalization has also contributed to the homogenization of the diet [14] and the standardization of processes of food transformation. This has been due to the need to adapt to international export standards and lower costs of food provision and access [15].

Although there are many studies that explain the link between globalization and the change in both diet and traditional food practices in the cities [6,16], the dynamics around agro-alimentary systems in the rural-urban gradient remain unexplored [8].

### 1.2. Diets and Traditions. The Cultural Value of the Traditional Mexican Diet

Food, apart from its nutritional value, is a key element of human identity. Food is culture when it is produced, through the farmers’ knowledge of the management of the field, crops and their varieties (agriculture), when it is transformed, using culinary techniques (cooking), and when it is consumed, bringing together economic, nutritional, and/or symbolic aspects [17].

In 2010, the Intergovernmental Committee for the Safeguarding of Intangible Cultural Heritage (ICH) of UNESCO, approved the candidacy of Mexico under the title: “Traditional Mexican cuisine—ancestral, ongoing community culture, the Michoacán paradigm” and it was included in the Representative List of the Intangible Cultural Heritage of Humanity [18]. This award is an acknowledgment of the agro-alimentary traditions of the country, the maximum expression of which can be seen in the Michoacán region. It highlights the value of the preservation of species and varieties that have their center of origin in Mexico (maize, beans, pumpkin, and chili), traditional growing methods, such as the *milpa*, and the culinary processes that transform the crop into food, such as nixtamalization, employing specialized tools, such as *metates*, stone mortars, and *comal* (prehispanic thin, disc-shaped device made from unglazed clay or metal used to cook maize tortillas) [19].

However, other studies have shown that traditional knowledge in general, and, specifically, culinary knowledge, which has been preserved mostly in farming systems in rural contexts since pre-Hispanic times, is eroding and transforming at an accelerated rate [20,21]. Currently, in Mexico, several institutions are working to strengthen the rural sector for the preservation of traditional agro-alimentary systems, from production to consumption [5]. However, a deeper analysis of the state of these systems is needed in order to respond to the challenges that exist both in the countryside and the cities and understand the relationship between them.

### 1.3. Maize Tortillas

Corn tortillas have been and continue to be the basic food of the Mexican diet and its main source of energy and protein. The center of origin and domestication of the *Zea mays* is Meso-America [22], specifically Mexico, where, currently, 59 native races and 300 varieties of maize are still preserved [23]. Despite the above-mentioned changes in food patterns of the population, this crop continues to be the basic pillar of the Mexican culture and food. With about 7.5 million hectares, it is responsible for most of the sown area in the country, which sets Mexico as the seventh largest producer and the fifth largest consumer of maize in the world [24]. Eighty-seven percent of the maize that is produced is white corn for human consumption, and 70.5% is cultivated under rainfed conditions [25]. The states with the largest volume of production are Sinaloa (19.5%), Jalisco (13.2%), and Michoacán (7.4%) [25].

It is estimated that in rural areas the consumption of maize is of about 274 kg/person/year [26] and that of tortilla is of up to 0.455 kg/person/day [27]. Although these numbers have not diminished, the way in which these are consumed has changed. Today, maize is present in a wide range of processed foods that do not require exhaustive labeling to indicate the type of maize that was used, nor its provenance. This has a direct effect on the loss of native races and the values associated with the traditional growing, transformation, and consumption practices. But what happens with tortillas?

Tortillas are a type of food made from nixtamalized maize which occupies a central place in the culture and daily diet in Mexico and Central America. Although the term “tortilla” was coined after the conquest (as a reference to the round-shaped Spanish omelet), the process of nixtamalization dates back to pre-Hispanic times—between 1200 and 1500 BC, according to the earliest evidence, found in South Guatemala [28].

From the náhuatl *nixtli* (ashes) and *tamalli* (dough), nixtamalized tortilla is made from just three ingredients: Maize, water, and limestone. The process begins by mixing three parts of water with 1% lime (Ca(OH)_2_) and one part of maize. The preparation is cooked for 30–90 min (the exact time depends on the type of maize, the moisture of the grain and the “strength” of the lime) [29]. The maize is then left to soak in the cooking water for 8–12 h, during which it increases from 12% to 100% of its final weight. After soaking, the cooking water, called *nejayote* is discarded and the maize is rinsed two or three times, without removing the pericarp or the germ. Cooking and rinsing should not be excessive, or the dough will lose its *correa* (colloquial term that refers to the elasticity of the dough, which prevents the tortillas from breaking) and become rubbery. On the other hand, if it is not rinsed enough, it can become coarse and develop a slightly bitter taste [29]. The result is a soft but cohesive dough that can have up to 45% of moisture [30,31].

Although maize is widely consumed worldwide, it is only nixtamalized in Meso-America. This process transforms the food physically, chemically and nutritionally through its alkaline action [32]. Some of its benefits include the increase in bioavailability of amino acids and greater content of phosphorus and calcium. It is also especially important in that it increases niacin availability [33].

However, not all maize tortillas are equal. Not even handmade comal tortillas, which, according to the collective imagery, are synonym with traditional tortillas [19]. The quality and nutritional content of tortillas depend on the decisions taken in the field and on the processes of food transformation. Michoacán is one of the states with the greatest diversity of native maize and the third state in maize production in the country [25]. However, it is important to remark that, although some authors claim that the richness of native maize has not diminished, the cultivated surface has, as it has been substituted with hybrid improved maize, introduced by large seed companies [34]. This diminishment has led to the lack of availability of native grain in some areas of the state (especially in urban centers) and in specific times of the year, as well as making it difficult for people to consume tortillas made with these types of maize. This is something that impacts the revalorization of both the agrobiodiversity and the traditional gastronomic culture and traditional diet.

The question that emerges from the points above would be: Is there, in the state of Michoacán, a gradient in the loss of traditions and quality in all aspects of the maize–tortilla system as we get away from rural environments and approach the city? In what ways has a medium-sized city such as Morelia, which is close to rural areas where native maize still exists and is cultivated and traditional transformation practices are maintained, been a victim to the influx of globalization (considering globalization as a phenomenon, that has influenced the homogenization of diets)?

Based on this, the objective of the present research is to analyze the main characteristics of the handmade comal tortilla system (from sowing to sale and/or consumption) in urban (Morelia), peri-urban and rural contexts in Michoacán, considering: (1) The type of seed and production, (2) the process of transformation, (3) channels of commercialization and purpose of sale, and (4) perceptions around the quality of the product.

## 2. Study Area and Methodological Approach

**Figure 1:**
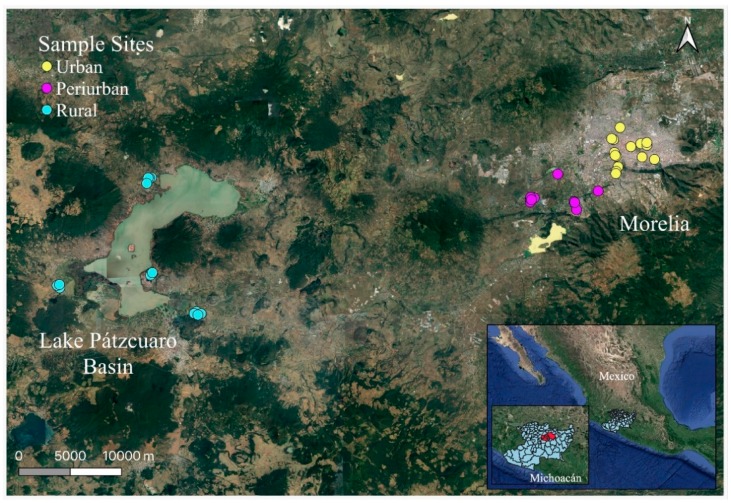
Map of the urban, peri-urban and rural areas of Morelia and the Lake Pátzcuaro Basin (LPB).

Three study areas were defined in the State of Michoacán, Mexico. One was an urban area located within the metropolitan limits of the city of Morelia. Another was a peri-urban area located southwest of the city of Morelia. The third study area was a rural area encompassing several communities located in the Lake Pátzcuaro Basin (LPB) (Figure 1). Throughout the study, results will be shown comparing the average of some areas with others. In some cases, the particularities observed in a specific area will be explained in detail.

Morelia, a medium-sized city, is the capital of the State of Michoacán. Around 600,000 people live in its metropolitan area [35]. Since the 1980s, it has shown an accelerated and heterogeneous population expansion [36]. The areas adjacent to the city, defined as diffuse urbanization, expanded periphery or peri-urban environments (in this study we will use the latter term) have particular characteristics, forming an imprecise gradient between rural and urban regions [37]. In the selected peri-urban area, crop fields coexist with urban settlements, and the inhabitants lead a mixed way of life. The selected rural area is located approximately 60 km from Morelia, on the shores of Lake Patzcuaro. Four rural centers were included, each with a population of less than 2500 inhabitants and belonging to different municipal entities: San Francisco Uricho (Mun. Erongarícuaro), Tzurumútaro (Mun. Pátzcuaro), San Pedro Cucuchucho (Mun. Tzintzuntzan), and San Andrés Tziróndaro (Mun. Quiroga).

The selection of the study areas was made based on the degree of similarity or difference in the production, processing, and sale of handmade comal tortillas (from now on HCT). The rural communities of the LPB have a long history of cultivating native varieties of maize using traditional methods and making HCT [38,39]. The peri-urban area is located along the main road that connects Morelia (urban area) with the LPB. There is thus a continuum in the access to and exchange of products and knowledge between urban and rural areas. It is important to note that other peri-urban areas in the city of Morelia (those harboring housing complexes for the upper classes, or those bordering the municipality of Tarímbaro to the north, the state’s main hybrid maize production area) are subject to different socioeconomic dynamics, depending on their geographical location or economic function.

**Figure 2:**
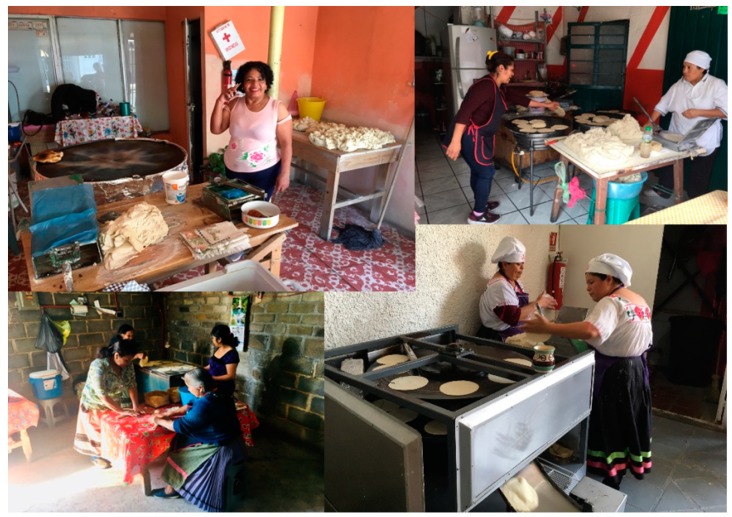
Handmade comal tortilla establishment in urban, peri-urban, and rural areas. Source: Authors.

Our object of study was the little establishments selling handmade tortillas, made and cooked by women on a comal. From now on, we will refer to these establishments as handmade comal tortilla establishments (HCTE). These establishments range from informal workshops (established by women who make tortillas in their homes, alone or helped by a family member, and who intermittently go out to sell them in their own community or in other communities) to more established companies that keep accounting and billing records and make use of hired labor (Figure 2). The criterion used to select the establishments was the use of the comal as a traditional tool for making tortillas, as opposed to the industrial tortilla shops that rely on mechanized processes.

We used a non-probability sampling method to determine the number of HMTE to be sampled [40]. A total of 41 semi-structured interviews were conducted in the three study areas. Fifteen interviews were conducted in HCTE in the urban area, 10 interviews were conducted in the peri-urban area, and 16 interviews were conducted in establishments or with people selling HCT in the four rural communities mentioned above. The semi-structured interviews were conducted during December 2018 and January 2019. They were divided into four main parts. The first part was linked to maize cultivation, the second to the techniques of maize transformation and tortilla processing. The third part was related to the tortillas’ sale strategies and their consumption and the fourth part were qualitative questions about the quality of their product and their life.

Peralta de Legarreta [21] proposes an approach for analyzing gastronomic culture based on four elements: (1) The link between man and land and natural resources, (2) the selection of the edible and the non-edible, (3) the culinary transformation of food, (4) consumption. Using this approach, the author analyzes the path or chain followed by food “from the land to the table”. The same approach is part of other analytic methods, such as the food value chains used by the FAO [41]. In the present study, we also differentiated a series of elements associated with the various activities carried out along the food chain. The first and second elements are related to the production and transformation of food, the third, unlike the work of Peralta de Legarreta [21], is related to the sale of the food product and, to a lesser extent, to its consumption, since the purpose of this study is to analyze establishments selling HCT.

## 3. Results

### 3.1. Type of Seeds and Tortilla Production Systems

ORIGIN and VARIETY: The results of our inquiries regarding the type of seeds used by HCTE show a gradient in terms of origin and variety of grains. In rural areas, tortillas made with local and native maize predominate, while in urban and peri-urban areas, most HCT are made with hybrid maize seeds produced in Sinaloa or Jalisco (Table 1). As has been noted by other authors, much of the production of native maize is not used, or only in a small percentage, by the Mexican agri-food industry. It is mainly produced for self-consumption and, to a lesser extent, for local trade [24]. This trend explains that 50% of the HCT sold in rural environments (and between 93% and 80% of those sold in urban and peri-urban environments, respectively) are made with hybrid maize from distant origins, especially during certain periods of the year. These data, however, come from four rural communities, Orozco-Ramírez et al. [42] determined that maize production in two of them was decreasing. Another regional study reported that 56% of the homemade and traditional tortillas consumed between 51% and 70% of the local native maize [43]. These data were confirmed 11 years later by Astier et al. (2019) in a study based on surveys applied in 19 rural communities. The strategy usually used by local HCTE in rural areas is to use local native maize until it runs out, and then buy maize from stores supplied from the Morelia Supply Center.

The Morelia Supply Center (MSC) is located in the northeastern periphery of the city. The MSC contains several maize-dispensing depots, most of them selling white hybrid maize from different origins. Sinaloa maize is highly demanded and appreciated for its homogeneity, whiteness, and consistency. This maize is produced under irrigation during the autumn-winter cycle, which makes it possible to supply the market when rainfed maize is scarce. Up to 90% of the maize used in Mexico is Sinaloa maize [44]. Its price remained unchanged between January and April 2019 at 5.4 MXN/kg in the MSC. Barca maize (Jalisco) is smaller than Sinaloa maize, but is valued for its homogeneity and for its lower cost of 5 MXN/kg (also unchanged between January and April 2019). Barca maize is rainfed maize, but due to the edaphoclimatic conditions of the area, it usually gives very satisfactory yields of approximately 7.11 t/ha [45]. Finally, Region maize (the term “Region” is used both in the MSC and by HCTE to refer to maize that comes from the northeastern area of the State of Michoacán and southeast of the State of Guanajuato), rainfed grown temporarily and characterized by its lower cost (4.7–4.9 MXN/kg) is not as preferred due to its low homogeneity and the presence of residues, compared to the previous two varieties. Astier et al. [38] made an interesting spatial analysis of the origin of the maize used to make handmade tortillas in the LPB.

COLOR: Tortillas made with white maize are generally preferred by consumers compared to tortillas made with blue or red maize, to the point of being impossible to find blue tortillas for sale in the peri-urban area. Blue tortillas (a distinctive sign of native maize), is only sold in three Morelia HCTE (in two of them, only upon request and subject to grain availability). Consumers of blue tortillas pay higher prices for what is considered an artisanal product. It is worth mentioning that in urban establishments selling blue tortillas (Table 2), the amount of blue tortillas sold does not exceed 6% of the total sales of tortillas.

In rural areas, blue tortillas are sold in small and informal establishments at the same price as white tortillas. These tortillas are mainly used for self-consumption, since selling them in rural areas does not generate an extra profit, as occurs in urban areas. This has been reported by other studies conducted throughout the country [46,47].

PRODUCTION SYSTEM: While the use of agrochemicals in maize production is not as widespread and intense as in the production of berries or vegetables, it is difficult to find farmers who grow 100% organic maize in Michoacán. Organic maize is produced on demand and is mostly destined for foreign markets in Canada and the US [48]. Some enterprises in the Purépecha Plateau, such as Marku Achekoren and Coyote Rojo, have been producing organic maize for decades. However, the present study identified an increasing (although still irregular) demand for products made with organic maize (especially tortillas) in urban areas of Michoacán, but supply is still scarce. In Mexico, organic production processes are regulated by the Organic Products Law, and export products must have the corresponding certification [49]. Participatory certification mechanisms exist at the local level, with greater flexibility in terms of time, lower costs and closer ties with producers, transformers, and consumers. Red Tsiri is an initiative working in the LPB that tries to improve the living conditions of producers of organic native maize and of the people who transform this maize into artisanal food products by selling directly to consumers or through short marketing channels in Morelia [50].

### 3.2. Transformation and Elaboration of Tortillas

The elaboration of HCT consists of the following main steps: (1) Nixtamalization, (2) grinding and use of metate, (3) pressing or *palmear* (“palmear” involves shaping the dough by hand until it forms a tortilla), (4) cooking on the comal. The type of maize grain used, the use or not of different flours, the type of fuel used for cooking and the elaboration of the tortillas can vary widely depending on the environment, urban or rural, and the natural resources (maize and firewood) available (Table 3).

NIXTAMAL: All rural HCTE prepare their own nixtamal using firewood and maize grains produced by themselves or bought from someone else. Nixtamalized dough is highly perishable and must be prepared daily for it to be fresh. Between 50% and 60% of urban and peri-urban HCTE buy the nixtamalized dough from third-parties to make the tortillas they sell. None of these HCTE were able to identify the origin or production process of the maize used to produce the dough they acquired. This ignorance is also shared by a large proportion of the consumers of the final product (tortillas) in Mexico, in contrast with the trends and the legislation regarding traceability found in the European Union (traceability is the ability to track the movements of a food product, from its production to its consumption, through all the links in the food chain, thanks to an identification and control system), although not all food products face the same requirements [49,51].

TECHNOLOGIES: As mentioned earlier, the traditional process of making tortillas involves manual and unsophisticated operations. Firewood (for cooking the nixtamal and heating the comal) is used in 75% of rural HCTE, the remaining 25% use gas. As we approached urban environments, the use of gas became more widespread: 40% of peri-urban and 100% of urban HCTE. We measured the investment cost of using firewood and gas to elaborate one dozen of HCT in three peri-urban and rural HCTE. The average cost in MXN was 2.37 and 3.83, respectively. It is essential to underline that in many rural households and establishments, firewood is collected, therefore it doesn’t represent a monetary cost. It is worth noting that 33% of the wood-burning stoves used in rural areas were Patsari-type stoves (the *Patsari* stove is the product of a participatory process of technological innovation. The design of this stove’s combustion chamber and tunnels is optimized, and its parts, including a metal chimney support and metal comales (pans to place the pots) are custom-designed for durability. The stove is constructed using a metallic mold to ensure that critical dimensions are maintained. The exterior structure is made of brick, and the internal body is made of a mixture of mud, sand, and cement. All of these materials are locally available, and the custom-made stove parts are also manufactured by small local industries), which improve the efficiency of firewood and pose lower health risks for the people who use them [52].

The use of a press, as opposed to the elaboration of tortillas by hand (*palmear*), is found in most HCTE, up to 57% of those located in rural areas. In urban and peri-urban areas, all HCT sold are made with a press (except for those sold in the markets by individual rural women and which are not the object of study of this work). Using a press saves time and effort, increasing the speed of production six-fold. The results of the present study indicate that consumers in all environments have a clear preference for pressed tortillas (thinner and more uniform). Novelo and García [53] recorded social differences in the consumption of tortillas at the beginning of the 20th century, with thin and white tortillas being consumed by the upper classes and thick and dark tortillas by indigenous people. Ortega et al. [54] studied a network of 89 women who made tortillas by hand (without the use of a press) in Oaxaca, which indicates, however, the preference for non-pressed tortillas in that region.

The artisanal grinding process that transforms nixtamalized maize into dough requires a pre-Hispanic utensil called “metate” (from the nahuatl *metatl*). Some people say that the taste of tortillas made with the use of a metate is different from those made with an electric mill. However, the high physical effort involved in the use of a metate led some researchers in the 1940s to consider indigenous women as “metate slaves” [55]. Nowadays, the metate is commonly used in rural areas for the final kneading of dough already ground in the mill. In our study, seven women used it (Table 3). Doña Micaela, from Cucuchucho community, told us about the usefulness of metate:

(...) “it is for the last kneading pass before throwing the tortillas on the comal...”

In the rural areas studied here, 87.5% of the HCTE took their dough to one of the community mills. There are usually between three to four mills in each community and the grinding prices vary widely, since they depend on individual agreements between the millers and the tortilla makers. The remaining 12.5% of the HCTE own a small electric mill. In peri-urban areas, of the HCTE that do not buy dough and prepare their own nixtamal, all use the mills belonging to the nearby industrial tortilla shops. In urban areas, that proportion rises to 60%. However, the remaining 40% of urban HCTE invested in their own mill in order to have control over the delicate grinding process.

Technology should not be at odds with the production of a quality final product, especially if technological tools are well designed, taking into consideration the needs and cultural preferences of users and consumers. Some authors have demonstrated the preference of rural households for native maize (especially for tortillas) and their resistance to change [38,42]. Other authors, mainly with an agricultural engineering background are in favor of changing the original ingredients of tortillas to make them more accessible and nutritious for the urban population, even promoting tortillas made from white sorghum [56]. Since the 1980s, some authors have advocated for gas to be used in all rural households [57], while others have shown evidence that as long as firewood is accessible, rural households will always prefer it over using firewood to prepare food products other than tortillas [58,59]. Some mechanized pressing processes have also incorporated a porous material similar to that of metates in order to preserve the traditional quality of tortillas.

USE OF FLOURS: The National Company for Popular Subsistence (Compañía Nacional de Subsistencias Populares - CONASUPO) was a Mexican parastatal company created in 1961 with the objective of increasing the levels of food intake (especially maize) of the most vulnerable sectors of society, through generalized and distributive subsidies. This program reduced by more than half the price of products, such as nixtamalized corn flour, making them more accessible, which, in turn, increased their consumption, especially in times of grain shortages. Today, although the price of nixtamal nixtamalized corn flour (three brands of nixtamalized corn flour have been identified in Michoacán: MASECA, MINSA and AgroMINSA. They all fulfill the same function, although some people point out small differences between them) has increased significantly (Maseca: 12 MXN/kg, Minsa: 10.5 MXN/kg, AgroMinsa: 10.75 MXN/kg (data obtained in March 2019 at the Morelia Supply Center (Appendix A)), its use is still widespread. It is estimated that in the rural areas of the LPB, machine-made tortillas use 30% of nixtamalized corn flour [38]. Many consumers know that machine-made tortillas include nixtamalized corn flour and other products (whitener, preservatives, enhancers, and softeners) (see Appendix A). The sanitary specifications and commercial information of nixtamalized dough, tortillas, and other products derived from corn are regulated by the official Mexican norm NOM-187-SSA1/SCFI-2002. The Mexican Corn Tortilla Foundation (Fundación Tortilla de Maíz Mexicana or FTMM), integrated by government sectors, international organizations, academics and other members of the civil society, has requested a review of the official Mexican norm on tortillas regarding the allowed additives, labeling standards, control of artificial flavorings and colors, etc. The stated purpose of these changes is to avoid risks to human health and promote a fairer market for traditional maize tortillas [60].

HCT appear, in the imagination of consumers, as a symbol of the value of artisanal and natural products. In general, consumers of HCT are more demanding in terms of taste and texture and feel that machine-made tortillas cannot meet their requirements. Our results indicate, however, that 50% of rural HCTE in the study area admit adding nixtamalized corn flour and/or wheat flour to the nixtamalized dough used to make HCT. This percentage decreases to 20% in peri-urban areas, while only two of the 15 HCTE studied in urban areas admit adding wheat or nixtamalized corn flour.

We found that, according to the interviewees, the use of wheat or nixtamalized corn flour for making HCT in rural areas can be explained by four main reasons:

(1) To fix a badly cooked nixtamal. If the nixtamal is overcooked, the dough loses consistency and becomes rubbery and watery. This can be fixed by adding wheat flour, which gives elasticity to the dough, and nixtamalized corn flour as a binder product.
(2) To fix a bad grind. If the stones of the mill have been recently cut, the dough can become *quebrada* or *martajada*, that is, with whole pieces of maize that have not been ground completely. In such a case, it is necessary to use a metate to finish the grinding. If, on the contrary, the stones of the mill are old and their marks have become blurred, the dough can turn out sticky, an effect similar to the one produced by overcooking the nixtamal.
(3) To increase the volume of the dough and make more tortillas. Due to the perishable nature of nixtamalized dough, it is important to calculate the volume of tortillas that will be possible to sell, so that the dough does not get spoiled. Sometimes, the opposite happens, more tortillas are sold than expected, and nixtamalized corn flour is mixed with the dough to make more tortillas and make a larger profit.
  “What MASECA flour does is for the dough to yield more tortillas instantly if the prepared nixtamal is not enough”
(4) To whiten the dough. The color of maize grains changes according to the variety. Tortilla establishments know that consumers have a greater preference for white tortillas, a fact that has been corroborated by other studies [61]. This drives tortilla makers to add wheat flour (totally white) to the dough and, in this way, whiten the tortillas made from it.

In addition to the reasons described above, which are closely linked to the particularities of the tortilla making process and the demands of the consumers, some people use nixtamalized corn flour to save time and make the elaboration of tortillas easier. This applies not so much to common tortillas, but to other products (mainly fried products) such as quesadillas and/or tostadas. Some of the interviewees indicated that they have become accustomed to using both corn and wheat flour because they have been doing so constantly since the implementation of the subsidy programs carried out by CONASUPO. It would be necessary to ask if, as Mitchell [15] suggests, these habits are the intended result of policies that aimed to alleviate a structural problem, such as food shortages, by implementing a temporary solution, such as subsidizing imported food products. Or if these habits are the result of short-term plans that did not take into consideration the possible consequences of the application of government policies. The paradoxes of state paternalism.

In urban environments, adding flours to the nixtamalized dough is more typical of industrial tortilla shops, since, according to some HCTE, nixtamalized corn flour is much more expensive than maize grain (12 MXN/kg vs. 5–8 MXN/kg). As indicated before, many urban consumers look for a differentiated product and are willing to pay more for tortillas made from 100% maize. This coincides with what Appendini [62] points out in one of his works when she talks about the phenomenon of industrialization through the generalized use of flour.

### 3.3. Commercialization and Consumption of Tortillas

VOLUME, PLACES, AND SALE PRICES: In urban areas, HCTE sell four times more tortillas than in rural areas, and twice as many as in peri-urban areas (Table 4). The figure in brackets in the third column corresponds to the average sales volume in urban HCTE, with the inclusion of an outlier establishment. The sales volume of the outlier establishment is similar to that of urban industrial tortilla shops, selling approximately 1000 kg of tortillas per day. This urban HCTE, however, produce various types of comal tortillas (in addition to other maize products such as tostadas or tortilla chips) and employ more than 15 workers. A higher sales volume allows them to diversify their products and marketing niches. This increase in sales volume as we approach urban areas goes together with increasing sophistication of HCTE, which start to be semi-permanent businesses. One passes from a rural context where a high number of women sell tortillas from their homes or go out to sell their tortillas in the community or in other communities, to established urban businesses with regular customers and fixed work schedules. The peri-urban environment shows a mixed picture, with cases of people selling tortillas from their homes or from informal street stalls, and cases of modest but settled tortilla shops.

Regarding the efficiency in the conversion of grain to tortilla, we hardly see any difference between rural and peri-urban areas (1.74 and 1.76, respectively). These are remarkably high values compared with the average of the Mexican tortilla industry, which varies between 1.3 and 1.5 [63]. Greater conversion efficiency was identified in rural environments for HCT to which nixtamalized corn or wheat flour is added (1.83), compared to tortillas to which no flour is added (1.68). The lowest efficiency was observed in urban HCTE (1.27), which contradicts the widespread belief that hybrid maize yields more tortillas than native maize. We did not find any difference between them, which coincides with the results of other studies in the region [43]. The conversion of dough to tortilla does not vary significantly, it is around 75% efficient in both urban and peri-urban HCTE.

The price of each kg of tortillas is slightly higher in rural areas (Table 4), with no significant differences between the price of those made with native, hybrid, blue, or organic maize. The most affordable price is observed in peri-urban HCTE. In the urban area, there are significant differences between the price of white and blue tortillas, the latter being 1.65 times higher (Table 4). It should be noted that HCTE that sell blue tortillas are located in areas of the city that have high socioeconomic levels [64], which confirms that this type of product tends to be sold as gourmet food accessible only to certain urban strata. In the three study areas, the price of HCT is higher than that of machine-made tortillas, which is set at 14.71 MNX/kg for Michoacán [65].

These results raise the question of what alternatives are available for the lowest socioeconomic strata of urban areas to access native maize tortillas or organic maize products, and what is the relationship with the high levels of obesity found in those areas. It would be very interesting to conduct a study on these issues and assess their relationship with food sovereignty.

CONSUMPTION AND WORK: The sale and consumption of HCT are associated with different factors, depending on the area in which they are studied, and reflect variable and heterogeneous conditions. In rural areas, they reflect the marginalization of agricultural work, as well as the importance of tortillas for the livelihood of rural families. In the rural environment, a high proportion of maize and tortillas is used for self-consumption, and the sale of HCT to be a way to obtain an additional economic income to access other necessary goods. It has been reported that maize tortillas also function as exchange currency among residents of rural communities [66]. In rural areas, the producers and the consumers of the corn-tortilla system coexist, exchange goods, dialogue and establish links between each other that go beyond mere commercial relationships. As other studies focusing on Mexican rural areas have pointed out, maize is the central element in the organization of the work carried out by rural inhabitants, their eating habits and the perception of their quality of life [23,67,68]. Our study corroborated this fact through the testimony of Doña Marta:

“Here in Uricho, we go to bed with maize and get up with maize”

As one approaches urban environments, the purpose of making and selling tortillas turns to economic gain and business consolidation, with the selling of tortillas as the only productive activity of each HCTE. In peri-urban environments, however, different situations coexist. Here, strategies associated with the rural environment (purchasing grains from local farmers because they are neighbors, exchange of food for specific jobs, etc.) coexist with strategies resulting from the pressure exerted by the greater supply of tortillas that can be found in the nearby city (lower selling prices, etc.). In these areas, HCTE are usually informal and unstable. These are areas where some poverty indicators are higher than in rural areas [64].

We must emphasize that in many cases, and in the three environments studied here, the production and sale of HCT do not generate enough income to be able to live solely from this productive activity. However, there are at least two reasons why this activity is maintained in cases in which it does not generate a continuous economic benefit: (1) First, economic need. As part of their daily domestic work, which includes making tortillas for the family, some women make more tortillas to sell them and have some money of their own. This activity does not generate a large or continuous income, but it helps relieve some economic difficulties. (2) The second reason is the need of feeling useful. This feeling is not only about receiving a financial reward, but also about the satisfaction of maintaining a clientele that values your work. Doña Socorro has a small stall in the Plaza de Tenencia Morelos, in the peri-urban area of Morelia, and she explains it this way: *“The most satisfactory part of my work is the dignity that earning a living gives to me.”*

Regarding consumption and nutritional issues, obesity and chronic diseases, such as type 2 diabetes among others, are strongly linked to high glycemic index diet [69]. Some authors have found that maize tortillas, produced from nixtamalized maize, present high levels of dietary fiber and resistant starch content associated with whole grains with lower glycemic index [70,71]. That corroborates the health benefits of nixtamalized tortilla consumption by improving food security.

### 3.4. Perceptions about the Quality of HCT

In some urban environments, it is usual to refer to comal tortillas as “traditional” or “artisanal” tortillas. These words confer a connotation of higher quality which, at the same time, gives the product an added value. Although quality is a social construction [46], and in many cases subjective, HCT are highly appreciated in towns and cities. A large market for them has usually existed in the country, providing a livelihood for “tortilleras” (women who make tortillas for sale) [52]. Lerner and Appendini [72] reported that many consumers look for “real and/or traditional” HCT rather than tortillas from commercial tortilla shops.

When we asked the people who run HCTE in rural areas about their perceptions of the terms “quality product”, “artisanal product” and “natural product”, they acknowledged not being familiar with these concepts and associated them indistinctly with flavor, texture, durability or the use of nixtamalized dough (without the addition of flour). They did not consider their product to be special or valuable. Most consider it a way to help them earn a livelihood, to survive. Doña Beatriz, from Cucuchucho, told us: *“Here, we only distinguish two types of tortillas, machine-made tortillas and handmade tortillas.”* Doña Beatriz also revealed the secret of a good tortilla, which coincided with what most of the people interviewed in rural areas told us: The secret is the nixtamal and tortillas are made from “pure maize”. Some people also mentioned that good tortillas should be “passed through the metate”.

In peri-urban areas, people are more familiar with marketing terminology, associating “quality product” with flavor and cooking and the term “natural product” with tortillas made with nixtamalized dough, however, the term “artisanal product” confused 100% of respondents. Not all urban establishments identified the terminology chosen in the study to define or classify their product, but, paradoxically, the three urban establishments that sell blue tortillas in Morelia did. Much of the scientific literature about tortillas focuses on the chemical and physical properties of a “good tortilla,” the most important being the breaking point, extensibility distance, and durability [46,73,74]. It is no surprise then that 75% of urban HCTE associate the term “quality product” with these properties. In this regard, Don Abdiel, the owner of a HCTE in Morelia declared: *“A soft tortilla does not break when bitten or bent, and swells again when reheated as if it were freshly made.”*

People who make and sell tortillas also have perceptions about their future and the continuity of this occupation. Generational replacement in the HCT business is in danger for 80% of the women in the rural areas, given that this activity is associated with lack of education, lack of opportunities, and poverty. These women also consider it a highly physically demanding job, although the hardest part is the uncertainty of being able to sell the product. None of the interviewed women in the rural sector wants this job for their children. However, these perceptions change drastically when the business is prosperous and innovative. In such cases, women say they would like their sons or daughters to continue with the activity. Some of the interviewed are proactive and see this activity as a life choice, revaluing its benefits, and they say that selling tortillas is a dignifying job.

## 4. Conclusions

Michoacán is one of the most culturally representative states of traditional Mexico. Proof of this are its agriculture, its richness in maize native races, and its gastronomy. However, the data obtained in the present study suggests that there is a gradient of loss of culinary traditions from rural to urban environments. Even rural areas have strayed away from traditional methods and ingredients.

The most contrasting characteristics observed suggest that in rural areas, HMCTE are conceived as livelihoods, while in urban areas HMCTE are established businesses with a prospect of continuity in the medium term. On the other hand, in many urban HCTEs, women are not necessarily in charge of the steps involved in the whole tortilla making process: Dough can be purchased, maize milled in industrial mills or as women, that are part of a manufacturing chain, are only in charge of one particular task. This phenomenon implies a loss of the ancestral knowledge of tortilla making as a whole.

The limited availability of native maize in urban environments sometimes makes it impossible to access native maize products and/or makes them more expensive. That is why almost 100% of HCT in these areas are made with hybrid maize from Sinaloa or Jalisco. In rural areas, native maize is mostly produced for self-consumption. Half of the rural HCTE use native maize for making the HCT they sell. Paradoxically, 50% of the HCT sold in rural areas contain MASECA and/or wheat flour, something that goes against what those same people defined as “quality tortillas”. The consumption of blue tortillas (which guarantees that they are made with native maize) is a luxury in urban areas, and a sign of backwardness in rural areas. There is no generational replacement perspective for tortilleras who make HCT due to the hardness of the work and its social connotations, associated with poverty. Tortilleras are one of the poorest socio-economic sectors, especially those from rural areas. These women will continue with this activity in the short future because of their limited access to other income-generating activities. For poor and marginal rural families, selling tortillas is an important source of income and daily currency, but generational replacement seems uncertain because nobody wants to condemn their daughters to poverty and/or a very physically demanding work.

Given that maize, nixtamalized corn flour, and fuelwood are the primary inputs needed to make HCT, the price of these determine its economic viability. Peri-urban and urban HCTE are more vulnerable to price increase and lack nixtamalized corn flour stocking because they depend almost 100% on gas and imported maize from other regions. Thus, rural HCTE were local maize and fuelwood are secured are less vulnerable and profitable. Firewood represents a relatively lower cost than gas for the ones that would need to purchase it.

There is a growing demand for HCT made of native maize and traditional nixtamal, however, in the peri-urban and urban sectors, innovation for making the whole process, with its four steps, easier and more efficient is urgent. Using appropriate technological tools for cultivating maize and cooking tortillas, such as *Patsari* or other improved cookstoves, could promote a more efficient and sustainable system of maize and tortilla production. Given the growing market of organic food, farmers could also find a secure market for the native maize they produce. This would allow indigenous landraces to be preserved and artisanal tortillas to be consumed in urban environments, preserving gastronomical traditions, securing local food security, and a providing tangible environmental, health, and economic benefits for rural and urban HCTEs.

## Figures and Tables

**Table 1:** Main characteristics of maize for handmade comal tortillas (HCT) in rural, peri-urban, and urban context.

Characteristic		Rural		Peri-Urban		Urban	
		% HCTE	Nº of HCTE	% HCTE	Nº of HCTE	% HCTE	Nº of HCTE
Origin	Local	50	8	30	3	13.5 *	2
	Regional	0	0	15	1.5	6.5 *	1
	Sinaloa/Jalisco	50	8	55	5.5	80	12
	Total	100	16	100	10	100	15
Variety	Native	50	8	20	2	7	1
	Hibrid	50	8	80	8	93	14
	Total	100	16	100	10	100	15
Color	White	90	14.5 *	100	10	97	13.5 *
	Blue	10	1.5 *	0	0	3	1.5 *
	Total	100	16	100	10	100	15
Production system	Organic	6	1	0	0	7	1
	Conventional	94	15	100	10	93	14
	Total	100	16	100	10	100	15

* In those establishments where several types of maize were used, the value was divided by the number of variables of that characteristic. Source: Semi-structured interviews.

**Table 2:** Characteristics of blue comal tortillas in the urban area of Morelia.

Urban Establishment (UE)	Purchase Price of Blue Corn (MNX/kg)	Sale Price of Blue Tortillas (MXN/kg)	Sales of Blue Tortillas (kg/week)	Blue Tortillas as Percentage of the Total Volume of Sales
UE No. 10	10	30	2.5	2
UE No. 11	13	40	25	0.35
UE No. 12	10	18	30	6

**Table 3:** Main features in HCT elaboration process.

Characteristic			Rural		Peri-Urban		Urban	
			% HCTE	Nº of HCTE	% HCTE	Nº of HCTE	% HCTE	Nº of HCTE
**Nixtamal**	**Corn**		100	16	40	4	46.7	7
	**Dough**		0	0	60	6	53.3	8
	**Total**		100	16	100	10	100	15
**Technologies**	**Heat source**	**Firewood**	75	12	60	6	0	0
		**Gas**	25	4	40	4	100	15
	**Total**		100	16	100	10	100	15
	**Grinding**	**Metate**	-	7 *	0	0	0	0
		**External mill**	87.5	14	100	10	60	9
		**Own mill**	12.5	2	0	0	40	6
	**Total**		100	16	100	10	100	15
	**Pressing**	**Hand**	43	7	0	0	0	0
		**Press**	57	9	100	10	100	15
	**Total**		100	16	100	10	100	15
**Use of flours**	**Yes**		50	8	20	2	13.3	2
	**No**		50	8	80	8	86.7	13
	**Total**		100	16	100	10	100	15

(*) Nº of HCTE that use *metate* as another source of grinding (not considered in the total sum). Source: Semi-structured interviews.

**Table 4:** Average values associated with the sale of tortillas in urban, peri-urban, and rural HCTE.

	Rural	Peri-Urban	Urban
**Sales volume (kg/week)**	71	107	260 (741)
**Efficiency Grain/Dough-Tortilla**	G-T: 1.74	G-T: 1.76	G-T: 1.27
		D-T: 0.73	D-T: 0.77
**Sales price (MXN/kg)**	18.5	16	Blue: 29.3
			White: 17.8

G: Grain, D: Dough, T: Tortilla.

**Figure A1:**
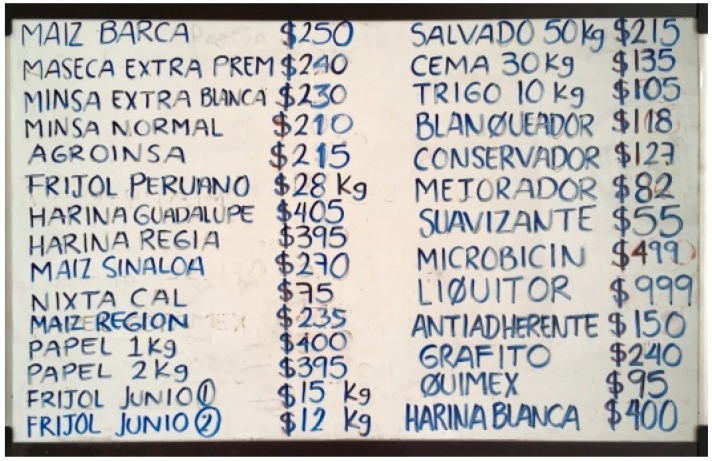
Prices of maize-related products for sale at The Morelia Supply Center (MSC) dispenser.
